# SOX9: Advances in Gynecological Malignancies

**DOI:** 10.3389/fonc.2021.768264

**Published:** 2021-11-22

**Authors:** Huan Chen, Yujie He, Xiangping Wen, Shihong Shao, Yujie Liu, Jinjin Wang

**Affiliations:** ^1^ Department of Obstetrics and Gynecology, Zhu Zhou Central Hospital, Zhuzhou, China; ^2^ Designated Ward, Zhu Zhou Central Hospital, Zhuzhou, China; ^3^ Department of Operation, Zhu Zhou Central Hospital, Zhuzhou, China; ^4^ Department of Pathology, The Affiliated Hospital of Qingdao University, Qingdao, China

**Keywords:** *SOX9*, ovarian cancer, cervical cancer, endometrial cancer, uterine carcinosarcoma

## Abstract

Transcription factors of the SOX family were first discovered in mammals in 1990. The sex-determining region Y box 9 belongs to the SOX transcription factor family. It plays an important role in inducing tissue and cell morphogenesis, survival, and many developmental processes. Furthermore, it has been shown to be an oncogene in many tumors. Gynecological malignancies are tumors that occur in the female reproductive system and seriously threaten the lives of patients. Common gynecological malignancies include ovarian cancer, cervical cancer, and endometrial cancer. So far, the molecular mechanisms related to the incidence and development of gynecological malignancies remain unclear. This makes it particularly important to discover their common causative molecule and thus provide an effective therapeutic target. In recent years, studies have found that multiple mechanisms are involved in regulating the expression of the sex-determining region Y box 9, leading to the occurrence and development of gynecological malignancies. In this review, we discuss the prognostic value of *SOX9* expression and the potential of targeting *SOX9* for gynecological malignancy treatment. We also discuss progress regarding the role of *SOX9* in gynecological malignancy pathogenesis through its mediation of important mechanisms, including tumor initiation and proliferation, apoptosis, migration, invasion, chemoresistance, and stem cell maintenance.

## 1 Introduction

Transcription factors of the SOX family were first discovered in mammals in 1990. The family is based on the conserved high migration group (HMG) box genes of the mammalian testis determinant *Sry*. Generally, proteins that contain an HGM domain and have 50% or higher amino acid similarity to the HMG are called SOX proteins (1). This family is subdivided into eight subgroups, from A-H, each subgroup contains 1–3 members (**Table 1**) (2). The SOX gene family encodes transcription factors that are conserved across species and participate in important developmental processes (3). The sex-determining region Y box 9 (*SOX9*) belongs to this family (4). According to the amino acid sequence of the HMG domain and transactivation and dimerization domain, *SOX9*, *SOX8*, and *SOX10* are grouped into the E subgroup (5). SOX9 protein contains an HMG box DNA binding domain that recognizes (A/T) CAA (T/A) G DNA sequences and controls the expression of target genes (6). It also contains a transcription activation domain located at the C-terminus (7) and plays an important role in inducing tissue and cell morphogenesis, survival (8), and regulation of many developmental processes (9); for example, embryonic development, lineage commitment, and stem cell maintenance (10). *SOX9* expression is elevated in numerous types of cancer, including lung, prostate, skin, brain, colorectal, pancreatic, and breast cancer (11–17). These studies indicate that *SOX9* acts as an oncogene in many cancers. Several additional studies have shown that *SOX9* is involved in the formation of cancer because an increase in its level is conducive to the transformation of stem cells. Furthermore, high levels of SOX9 are related to the tumor grade, poor prognosis, and poor survival of some types of cancer (18). In rodents, the expression of *Sry* initiates the downstream signal cascade by directly regulating *SOX9 (* 19). However, key *SOX9* regulatory genes in most human tissues and cancers have yet to be established; it maybe that they are cell type and developmental stage specific (9). It has been noted that *SOX9* has the opposite function of promoting and inhibiting proliferation, which indicates that its functions in proliferation are diverse and vary according to different environments (20). Therefore, the expression and function of *SOX9* alters in different human cancers mainly by regulating the activity of cancer stem cells (CSCs), and as a tumor suppressor under certain circumstances (18).

**Table 1:** SOX family and subgroup member.

Subgroup	Member
SoxA	SRY
SoxB1	SOX1, SOX2,SOX3
SoxB2	SOX14,SOX21
SoxC	SOX4, SOX11 and SOX12
SoxD	SOX5, SOX6 and SOX13,
SoxE	SOX8, SOX9 and SOX10
SoxF	SOX7, SOX17 and SOX18
SoxG	SOX15
SoxH	SOX30,

SOX family is subdivided into 8 subgroups, from A-H, each subgroup contains 1-3 members.SOX9 belongs to SoxE subgroup.

Gynecological malignancies are tumors that occur in the female reproductive system and seriously threaten the lives of patients (21). Common gynecological malignancies include: ovarian cancer (OC), cervical cancer (CC), and endometrial cancer (EC). To date, the molecular mechanisms related to the incidence and development of gynecological malignancies are still unclear. This makes it particularly pertinent to discover their common causative molecule and to provide an effective therapeutic target. In recent years, studies have found that multiple mechanisms are involved in regulating the expression of the *SOX9* gene, leading to the occurrence and development of gynecological malignancies. In this review, we discuss the prognostic value of *SOX9* expression and the potential of targeting *SOX9* for gynecological malignancy treatment. We also discuss progress regarding the role of *SOX9* in gynecological malignancy pathogenesis through its mediation of important mechanisms, including tumor initiation and proliferation, apoptosis, migration, invasion, chemoresistance, and stem cell maintenance.

## 2 The Structure of *SOX9*

**Figure 1:**
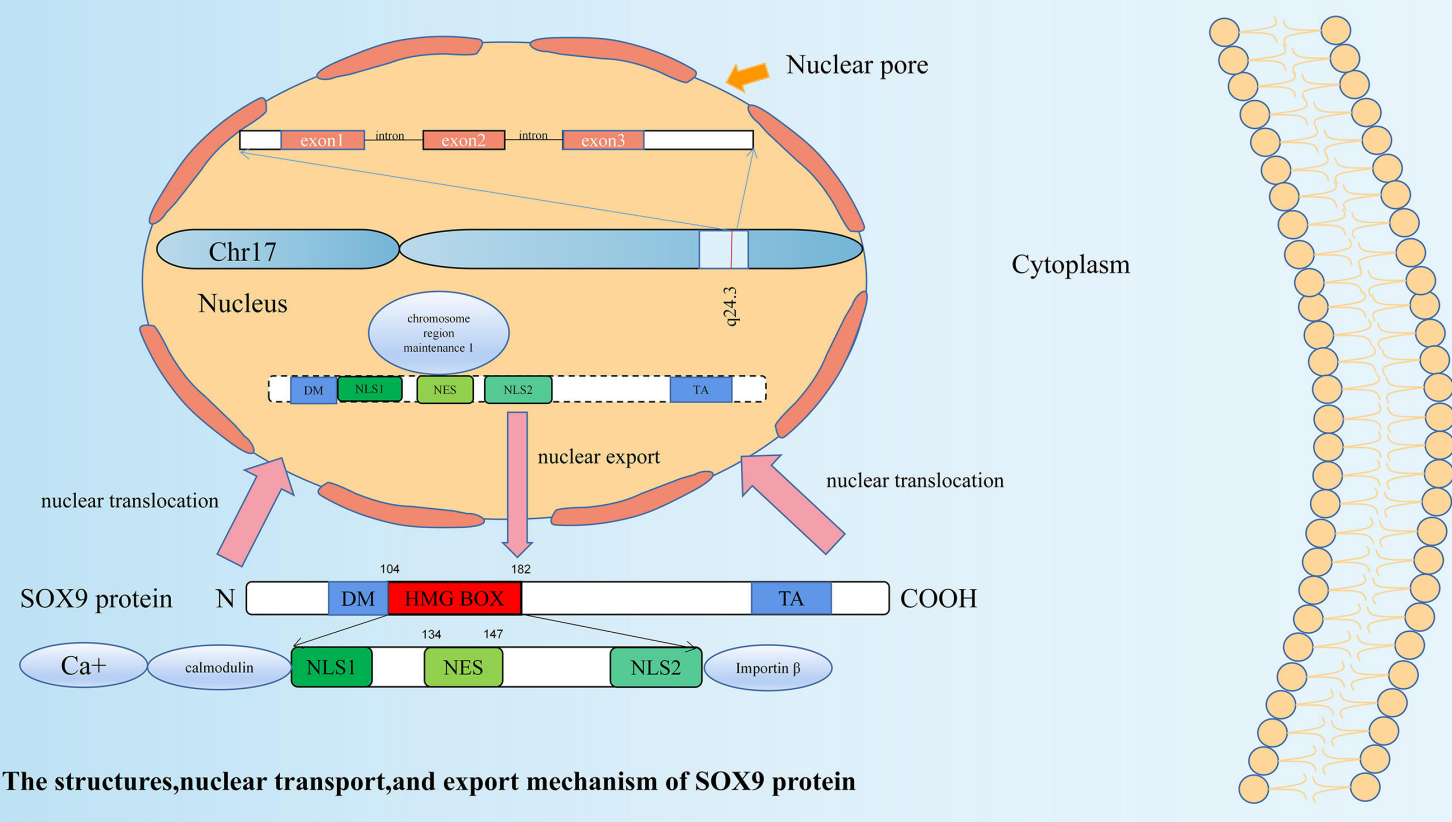
The structure, nuclear transport, and export mechanism of SOX9 protein. *SOX9* has three exons, two introns and an open reading frame (ORF). SOX9 protein is a polypeptide containing 509 amino acids. A total of 79 amino acids from positions 104 to 182 of the polypeptide chain constitute the HMG box. In addition to the HMG domain, SOX9 has two other functional domains: a dimerization domain (DIM) and a transactivation domain (TA). Its HMG domain contains two nuclear localization signal (NLS) sequences and one leucine-rich nuclear export signal (NES) sequence. NLS located at the N-terminus promotes nuclear translocation of *SOX9* by binding to calmodulin activated by calcium ions. NLS located at the C-terminus interacts with importin β to form a complex, which then, *via* the RAN GTP-dependent pathway, mediates *SOX9* to complete nuclear transport through the nuclear pore. The NES sequence located between the two NLS sequences can interact with chromosome region maintenance 1 to mediate the nuclear export of *SOX9*.

*SOX9* is located at 17q24.3~q25.1 in humans. It is 3934 bps in length, has three exons, two introns, and an open reading frame (ORF). Its coding product is a polypeptide containing 509 amino acids (22). A total of 79 amino acids from positions 104 to 182 of the polypeptide chain constitute the HMG box, and it has 71% homology with the HMG box of the *Sry* gene (23). Its HMG domain contains two nuclear localization signal (NLS) sequences (24) and one leucine-rich nuclear export signal (NES) sequence (25). These NLS sequences and the NES sequence can be activated by different pathways, enabling *SOX9* transcription factors to be present in both the nucleus and cytoplasm, and allowing regulation of the expression of related genes. NLS located at the N-terminus promotes nuclear translocation of *SOX9* by binding to calmodulin activated by calcium ions, which can be inhibited by calmodulin specific antagonists (26). NLS located at the C-terminus interacts with importin β to form a complex (27), which then, *via* the RAN GTP-dependent pathway, mediates *SOX9* to complete nuclear transport through the nuclear pore (28). Whereas, the NES sequence, located between the two NLS sequences, can interact with chromosome region maintenance 1 to mediate the nuclear export of SOX9 (25). SOX9 is a nuclear protein (24), and it is generally believed that *SOX9* nuclear expression has its function (29). The structure and nuclear transport and export mechanisms of *SOX9* protein are shown in **Figure 1**.

## 3 OC

The World Health Organization (WHO) categorizes ovarian tumors according to their histological differentiation, defined as epithelial, sex-cord stromal, and germ cell tumors, of which epithelial ovarian tumors constitute the most common type of OC (30). According to the latest data, OC ranks eighth in the world for female cancers, with 313 959 new cases and 207 252 deaths in 2020 (31).

### 3.1 Sex-Cord Stromal Tumors (SCST)

SCST account for 10% of OC, ranging from benign to low-grade malignant, and may be differentiated into male (Sertoli and Leydig cells) or female (granulosa and theca cells) structures. The main types of SCST include granulosa cell tumor, Sertoli-Leydig cell tumor, and steroid cell tumor (32).

#### 3.1.1 Sertoli-Stromal Cell Tumors (SSCT)

*SOX9* protein is a downstream effector of the testicular determinant *Sry*; it plays a core role in Sertoli cell differentiation and testicular cord formation after its translocation to the nucleus. *SOX9* is expressed in the testis, particularly in Sertoli cells, while it is inactive in follicular cells of the ovary. Ovarian SSCTs also show testicular differentiation. Therefore, in 2004, Kato et al. examined *SOX9* mRNA expression of two SSCT samples. One case was well-differentiated SSCT and the other was a Sertoli-Leydig tumor of intermediate differentiation. The results showed that *SOX9* mRNA was expressed but *Sry*-specific sequences were not detected in either specimen, while in normal testes and ovaries of the control group, *SOX9* was not expressed (33). This suggests that *Sry*-independent *SOX9* expression may be related to Sertoli cell differentiation in SSCT and its potential use as a marker for differential diagnosis. Subsequently, to investigate the role of *SOX9* in the histological differential diagnosis of ovarian Sertoli cell tumors from other tumors, Zhao et al. performed *SOX9* immunohistochemical staining on 152 ovarian tumors, including 36 pure Sertoli cell tumors, 38 endometrioid borderline tumors, 26 well-differentiated endometrioid carcinomas, 13 sertoliform endometrioid carcinomas, and 39 carcinoid tumors. Results included: (1) A spectrum of immunostaining intensities was shown in all tumor categories, and no significant diagnostic trends were found in any group. (2) The differences in mean immunohistochemical composite scores between the Sertoli cell tumors and the other four tumor categories were not statistically significant. (3) The extent score for sertoliform endometrioid carcinoma showed that *SOX9* was found in much lower numbers of cells than other tumor types (34). Therefore, the significance of the expression and function of *SOX9* in ovarian tumors remains unclear. By 2008, Papanastasopoulos et al. had performed immunohistochemical expression of *SOX9* through the regulation of prostaglandin D Synthase (*Pdgs*) in four primary SCST samples, including two Sertoli-Leydig cell tumors (one well-differentiated and one poorly differentiated) and two granulosa-cell tumors. As in the study by Kato et al. (33), *SOX9* expression was present in all four specimens and restricted to tumor cells containing a Sertoli-cell component. In contrast, it was not expressed in tumor cells with Leydig-like cell and granulosa-like cell components. The site of expression was mainly cytoplasmic staining, with some nuclei showing positive. *Pdgs* was expressed in both Sertoli-like cell and granulosa-like cell components, but not in Leydig-like cell components (35).

This further demonstrates that *Sry*-independent *SOX9* expression is associated with Sertoli cell differentiation. They also elucidated the mechanism of *Sry*-independent *SOX9* upregulation: *Pgds* produce prostaglandin D2 (*Pgd2*), which is necessary and sufficient for recruiting non *Sry* expressed cells to be able to express *SOX9* and differentiate into Sertoli cells.

These studies suggest that *SOX9* may be involved in Sertoli cell differentiation in SSCT, and that it is mainly expressed in the cytoplasm; but whether it helps to distinguish OC subtypes remains to be further investigated.

#### 3.1.2 Granulosa Cell Tumors (GCT)

In 2010, Kalfa et al. examined *SOX9* immunohistochemical expression in six cases of juvenile GCT with hyperandrogenism and 24 cases without hyperandrogenism; they found that there was no correlation between its expression or nuclear localization and hyperandrogenicity (36). In a Foxo1/3 dKO murine model for adult GCT, *SOX9* was not present in normal granulosa cells; however, it was present in the nuclei of some granulosa cells with follicle-like structures that were apparently missing from the oocytes, as well as in the nuclei of many tumor granulosa cells located within tubular structures. *SOX9* expression in mice GCT may reflect more of a shift to an epithelial-like phenotype than a transition to Sertoli-like cells (37).

These findings suggest that *SOX9* is predominantly expressed in the nucleus in granulosa cell tumors and may be a potential key to distinguishing this from other subtypes of SCST.

### 3.2 Epithelial Tumors

Ovarian epithelial tumors are the principal pathological type of OC, accounting for 85%–90% of OC (38). *SOX9* has been relatively well investigated in this type of tumor. Several studies have found that *SOX9* is more highly expressed in OC tissues than in normal ovarian tissues (39–41), adjacent normal counterparts (42, 43), or even effusions and solid metastases (44). Therefore, the role of *SOX9* in the prognosis of OC patients has been investigated.

#### 3.2.1 *SOX9*, Progression-Free-Survival (PFS), and Overall Survival (OS)

The first study to identify a correlation between *SOX9* and prognosis was by Raspaglio et al. There was no association between *SOX9* cytoplasmic expression levels and survival outcomes, but OS was significantly shorter in patients with increased nuclear expression of *SOX9*. The possible mechanism is that *SOX9* allows OC cells to survive under hypoxic conditions by activating the expression of βIII-tubulin proteins (45). Subsequently, while studying the role of *SOX9* in hepatocellular carcinoma, to examine the prevalence of *SOX9* expression as a prognostic factor in other cancers, Richtig et al. analyzed unbiased published datasets for breast, ovarian, lung, and gastric cancers. They found that high *SOX9* expression levels were a strong predictor of PFS in OC (46). Similarly to the first study, Sherman-Samis et al. found that higher *SOX9* mRNA levels were correlated with shorter OS in a univariate analysis, and a trend towards worse OS was observed for high *SOX9* levels in chemotherapy-naïve effusions (44). All these suggest that higher *SOX9* levels may be an independent prognostic indicator in ovarian epithelial tumor patients. In the same study, they also found that silencing 70% of *SOX2* and *SOX9* significantly reduced the MMP activity of OVCAR3 cells in gelatin-impregnated SDS gels, invasion in the Boyden chamber system in matrix-encapsulated filters, and motility in wound healing assays (44). These findings further suggest that *SOX9* may play an important role in the invasive process of ovarian epithelial carcinoma with nuclear expression. Therefore, it could be a potential therapeutic target for OC. The questions remain: what mechanisms are involved in the regulation of *SOX9* in OC? and in what ways does its role manifest itself?

#### 3.2.2 Postranscriptional Regulation of SOX9 Expression by MicroRNAs

MicroRNAs (miRNAs) are a class of small non-coding RNAs that have important roles in gene regulation (47). In mammals, miRNA regulatory roles have been identified in many areas of biology, which emphasizes that miRNAs are an exciting new class of therapeutic targets with a wide range of applications (48). It was found that *p70^S6K^*, a key kinase controlling the translation of target mRNAs, directly bind miR-145 and increase the expression of *Twist* and *SOX9*, thus promoting the formation of multicellular spheroids (MCS). Whereas, the key to extensive peritoneal dissemination and malignant ascites in OC is the ability to form MCS (49). Dexmedetomidine (DEX), an adjuvant analgesic during cancer treatment, has a dose-dependent suppression effect on OC cells line growth. It upregulates miR-185 expression, which further suppresses *SOX9* expression, and then causes inactivation of the Wnt/β-catenin signaling pathway, thus inhibiting OC growth and development (50). Xiao et al. found that miR-34c reduced chemoresistance of OC cells to DDP by inhibiting *SOX9* expression through the β-catenin signaling pathway (42). Meanwhile, MiR-30a-5p increased the sensitivity of OC cells to DDP by downregulating *SOX9 (* 41). All these findings suggest that the upregulation of some miRNAs can inhibit *SOX9* expression, thereby limiting the growth of OC cells or forming MCS and increasing the sensitivity of OC cells to chemotherapy, and could therefore be used as a more precise treatment target. Similar findings have been found in studies of other members of the SOX family: MiR-138 inhibits OC cell invasion and metastasis by targeting *SOX4* and *HIF-1α (* 51); MiR-223-3p increases OC cell proliferation and invasion by decreasing *SOX11* expression (52); MiR-492 promotes migration, invasion, and EMT capabilities *via SOX7* in OC (53). These findings reinforce the therapeutic role of the SOX family in tumors.

#### 3.2.3 Regulation of SOX9 Expression by Long Non-Coding RNAs

Competing endogenous RNA (ceRNA) networks are increasingly found to play an important role in carcinogenesis (54); it links the function of protein-coding mRNAs to that of non-coding RNAs (55). LncRNAs as endogenous RNA competitive RNAs (ceRNAs) can interact with miRNAs, and miRNAs participate in the regulation of target gene expression by binding to the 3UTR of target mRNAs (56).

To our knowledge, there are only three studies on the regulation of *SOX9* by ceRNA network in OC. The first study showed that LINC00115 binds miR-30a then upregulates *SOX9* and the Wnt/β-catenin pathway, thereby enhancing the stemness of ovarian CSCs and preventing apoptosis (57). The second study showed that LINC01132 acts as an oncogene in epithelial ovarian cancer (EOC) cells by controlling the miR431-5p/*SOX9* axis to increase migration and invasion of EOC cells (43). The third study showed that LINC00284 promotes serous ovarian carcinoma (SOC) initiation and progression through the *SOX9*-LINC00284-miRNA-195/497-5p-mRNA network (40). Taken together, the ceRNA network regulates the expression of *SOX9* and plays an important role in ovarian carcinogenesis, development, maintenance of cancer stem cell properties, anti-apoptosis, migration, and invasion of cancer cells. These also provide a new direction for the treatment of OC.

#### 3.2.4 *SOX9* and Chemotherapy Resistance

Chemotherapy resistance is a leading barrier to OC treatment, so overcoming chemoresistance is an important goal in OC treatment (58). Studies have shown that *SOX9* is involved in this process. *SOX9* silencing can slightly sensitize OC cell lines to paclitaxel and cisplatin (DDP) (45). It has also been found that exosome-carried miRNAs regulating *SOX9* are involved in the progression of chemotherapy resistance in cancer cells (59). MiR-34c reduces chemoresistance of OC cells to DDP by inhibiting *SOX9* expression through the β-catenin signaling pathway (42). MiR-30a-5p increases the sensitivity of OC cells to DDP by downregulating *SOX9 (* 41). The results of Shang et al. showed that *SOX9*, a key super-enhancer regulatory transcription factor (TF) target, is not only required for maintaining the cisplatin-resistant state but is also essential for acquiring the cisplatin-resistant state in OC cells. There is significant sensitivity to cisplatin in cisplatin-resistant SKOV3 or OVCAR4 cells with depletion of *SOX9*. The mechanism is likely to involve the binding of *SOX9* to the wnt5a loci in the WNT/β-catenin pathway (60). These studies suggest that rescuing miRNA expression in OC to inhibit *SOX9* and WNT/β-catenin signaling activation might provide a promising strategy for dealing with chemoresistance of OC to DDP. The more powerful evidence is the study of Sherman-Samis et al. They found that high mRNA and protein cytoplasmic expression of *SOX9* was significantly associated with intrinsic chemoresistance, with a trend of higher expression in patients with poor chemotherapy response to first-line chemotherapy (44). These studies suggest that *SOX9* expression can be used as a judgmental indicator prior to chemotherapy selection and also to increase patient sensitivity to chemotherapy by inhibiting its expression.

#### 3.2.5 Functional Roles of SOX9 in Cancer Stem-Like Cells

CSCs are some stem-like cells in cancer that have the ability to self-renew and differentiate (61). Characteristics of CSCs include self-renewal, the ability to form spheres and colonies in soft agar, tumor formation in nude mice, and differentiation into stem and non-stem cells *in vivo (* 62). They are thought to be the underlying cause of recurrence, metastasis, and drug resistance in many cancer types (63). Study has identified that *SOX9* is a proliferation and stem cell factor in hepatocellular carcinoma (46), and it has the same role in OC. *ST6Gal-I* induces upregulated expression of key transcription factors such as *SOX9* and *Slug*, which contribute to the growth of spheroids. *ST6Gal-I* is the main enzyme responsible for nglycan α2-6sialylation on selected glycoproteins, and its activity regulates cell adhesion, migration, differentiation, and survival (64). By phosphorylating glucose to glucose-6-phosphate (G6P), hexokinases (HKs) catalyze the first irreversible enzymatic step in glucose metabolism. Through ‘focal adhesion kinase (FAK)/extracellular signal-regulated kinase (ERK1/2) activation-induced matrix metalloproteinase 9 (MMP9)/NANOG/SRY-Box 9(*SOX9*),’ overexpression of HK2 in OC controls lactate production and promotes metastasis and stemness of OC cells (65). As mentioned previously, LINC00115 is involved in ovarian CSC stemness through the miR30a/*SOX9* axis (57).

**Figure 2:**
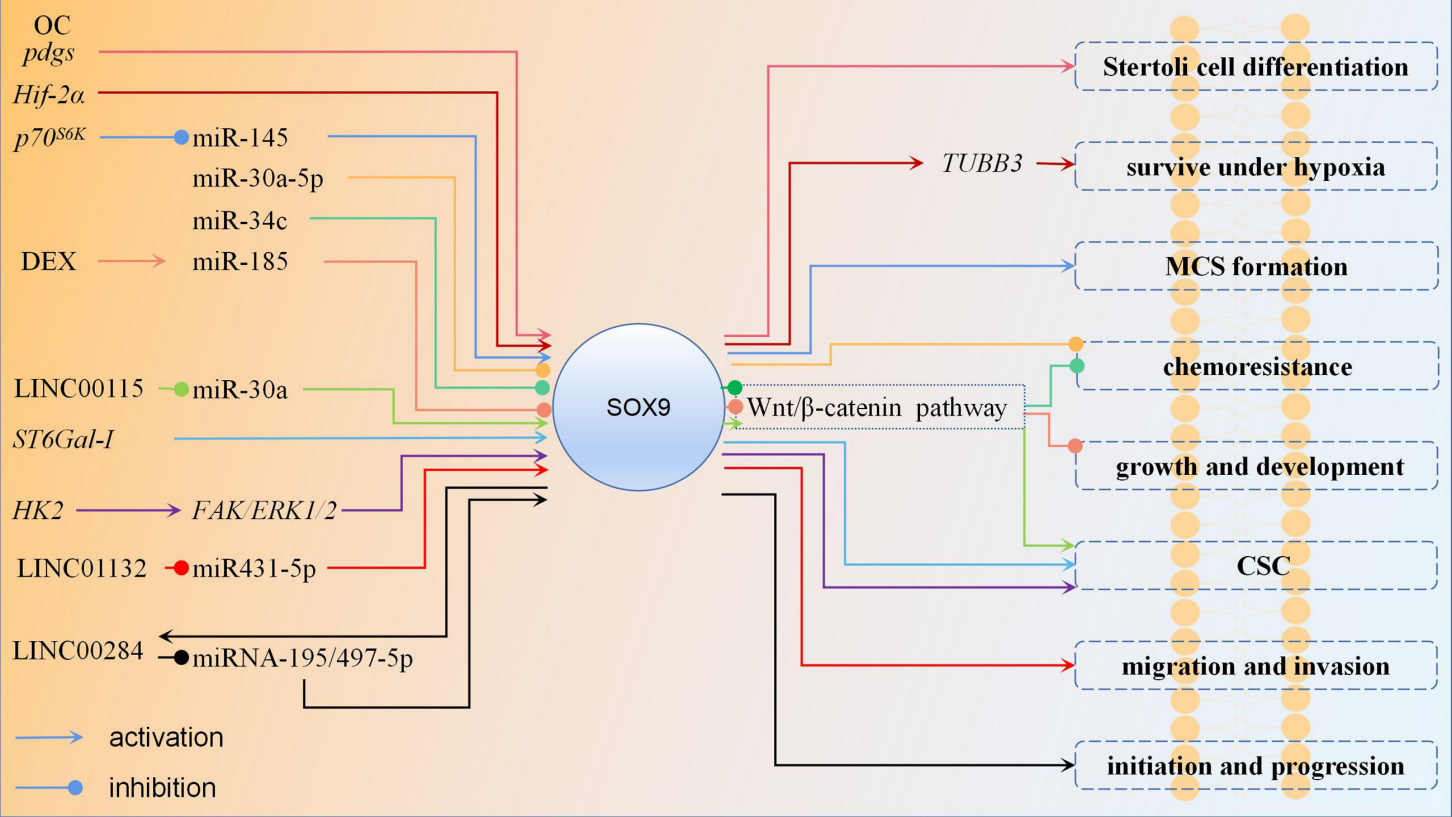
The regulation mechanism of *SOX9* in ovarian cancer. In most studies of ovarian cancer, *SOX9* plays an oncogene role. Upregulation of *SOX9* expression promotes Stertoli cell differentiation, ovarian cancer cell survival under hypoxia, MCS formation, chemoresistance, growth, development, CSC stemness, migration, invasion, initiation and progression. Promoting the expression of certain miRNAs can inhibit the expression of *SOX9* and thus suppress ovarian cancer. Wnt/β-catenin pathway is also an important pathway, and downregulation of SOX9 expression can inhibit it then suppress ovarian cancer chemoresistance, growth, development, CSC stemness. Each color represent a regulation mechanism of *SOX9*.

Most studies suggest that upregulated expression of *SOX9* in OC promotes cancer cell proliferation, metastasis, etc. However, there is one study that shows the opposite results. *Pgd2* is produced by the enzyme *Pgds*, which is synthesized in many organs and acts as a signaling molecule in the regulation of various biological processes. It was found that *Pgd2* acts as an autocrine factor to induce *SOX9* expression and nuclear translocation. *Pgds* and *SOX9* are highly expressed in proliferatively active solid tumors, while the anti-proliferative effects of *Pgd2*/*SOX9* are observed *in vitro*. The possible reason for this may be the induction of apoptotic gene expression owing to the conflict between proliferation and differentiation within tumor cells (39). However, why *SOX9* plays the opposite role *in vitro* study, needs to be further investigated. The regulation mechanism of *SOX9* in OC is shown in **Figure 2**.

## 4 Cervical Cancer

CC is one of the most common malignant tumors in female patients. Although early detection of CC improves the prognosis, it is still the fourth most common cause of death in women and ranks first in gynecological malignancies, with 604 127 new cases and 41 831 deaths in 2020 (31). Therefore, it is necessary to explore the detailed mechanism of CC progression and to identify new effective treatment targets.

### 4.1 Evidences for SOX9 Functions as Tumor Suppressor

While most studies in OC suggest an oncogene role for *SOX9* in OC, several studies in CC suggest *SOX9* is a suppressor. Methylation of CpG islands located in gene promoters often leads to transcriptional silencing (66). DNA methylation contributes to various diseases, particularly human cancers (67). The DNA methylation level of *SOX9* gradually increases in normal cervical tissues, CIN I, CIN II-III, and CC tissues. This potentially provides a valuable molecular biomarker for CC screening (68). However, the investigators did not assay these specimens for *SOX9* expression, therefore, the effect of methylation on the expression of this gene could not be judged. Subsequently, an immunohistochemistry study found that the protein expression of *SOX9* decreased gradually in normal cervical tissues, CIN III, and CC tissues. Overexpression of *SOX9* could inhibit the proliferation of CC cells *in vivo* and tumor formation of CC cells *in vitro*. The possible mechanism is that *SOX9* directly transcriptionally activates p21 by binding to the p21 promoter sequence of CC cells, then cells arrest at the G1/S phase transition point (69). These findings all suggest that *SOX9* is a tumor suppressor gene in CC.

### 4.2 *SOX9* and Single Nucleotide Polymorphisms (SNP)

Gene mutations, such as SNP, have been shown to affect cancer susceptibility in recent years (70). Japanese scholars performed genome-wide control studies of five gynecological diseases using data from 46 837 subjects (5236 fibroids, 645 endometriosis, 647 OC, 909 endometrial cancer, 538 CC, and 39 556 shared female controls) from the Japanese biobank project. They found that the locus rs140991990: A>G which is located at *SOX9* is associated with the pathogenesis of CC (71). As far as we know, this mutation is located in the intron of *SOX9* (https://www.ncbi.nlm.nih.gov/snp/?term=rs140991990). However, intron mutation will only cause changes in the composition and structure of intron transcriptional mRNA, and then affect the maturation and processing of mRNA, finally affecting the protein. The pathogenic risk of SNP in the intron region is significantly lower than that in the gene coding region and regulatory region. Therefore, the role of this *SOX9* SNP in CC needs further study.

### 4.3 *SOX9* and Oncogenes

**Figure 3:**
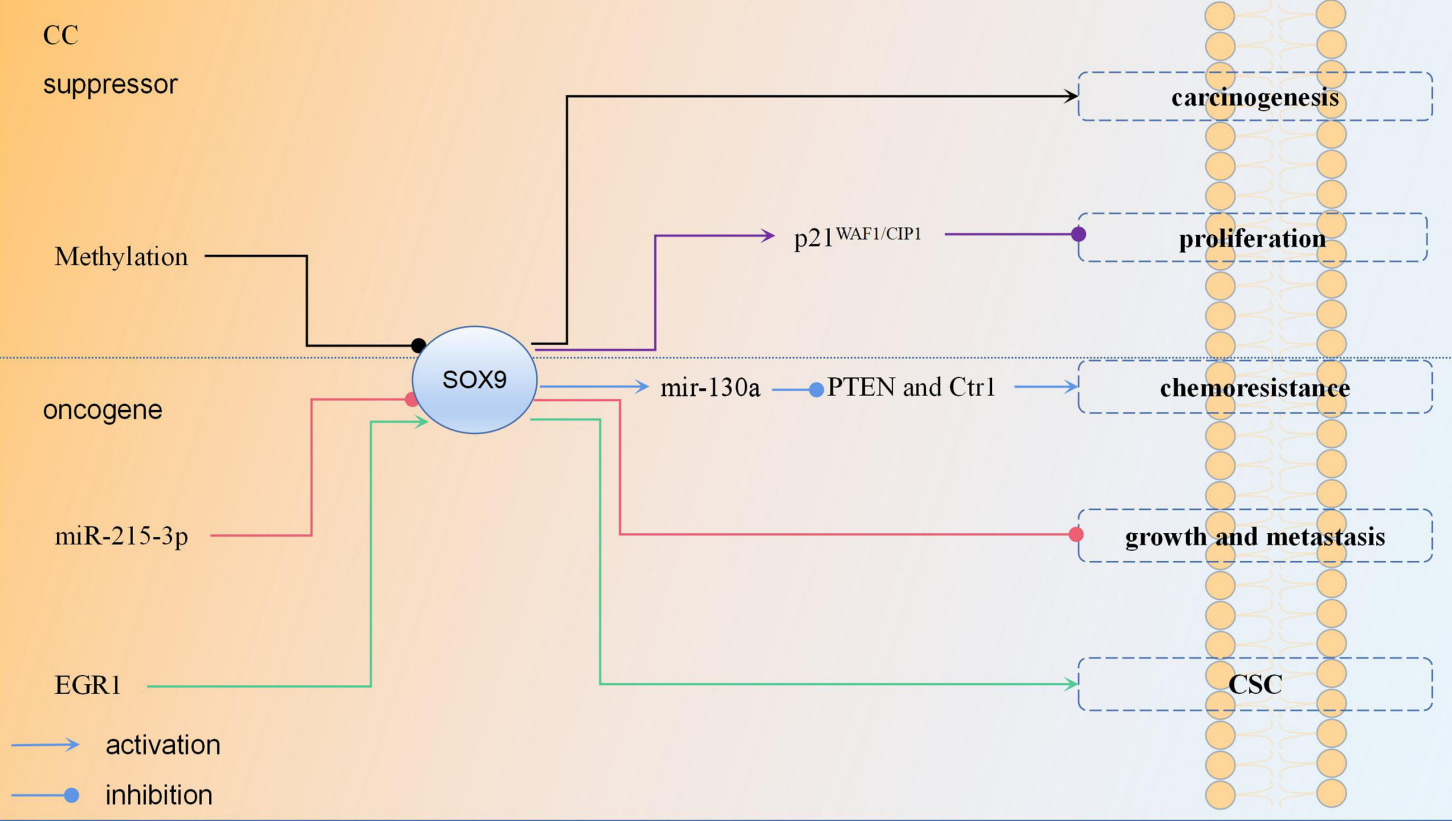
The regulation mechanism of *SOX9* in cervical cancer each color represent a regulation mechanism of *SOX9*.

Similar to the results of its OC research, the role of *SOX9* in CC also has the opposite research results, suggesting that *SOX9* plays an oncogene role in CC: (1) Compared with DDP-sensitive tissue, *SOX9* was significantly upregulated in DDP-resistant tissues; (2) Knockdown of *SOX9* sensitized CC cells to DDP; (3) *SOX9* activated the expression of miR-130a by binding to the promoter region of miR-130a, thereby inhibiting the expression of *PTEN* and *CTR1*, the downstream genes of miR-130a, and finally promoting the chemoresistance of CC cells to DDP (72); (4) *SOX9* was overexpressed in CC in comparison with the control of normal tissues; (5) Downregulation of *SOX9* inhibited the growth and metastasis of CC cells, and the upregulation of miR-215-3p inhibited the expression of *SOX9*, thereby inhibiting the growth and metastasis of CC cells *in vivo (* 73); (6) By inducing *SOX9* expression, *EGR1* facilitated CC stemness then promoted the proliferation and invasion of CC cells; (7) There was a significant difference in the 5-year OS rate of patients between low and high nucleus expression of *SOX9;* high *SOX9* expression was significantly associated with lower OS rate (74). These studies suggest that upregulation of *SOX9* is involved in the formation of CC, chemotherapy resistance, tumor cell stemness, metastasis, and invasion; therefore, high *SOX9* expression can be a biomarker for predicting the poor prognosis of CC. The regulation mechanism of *SOX9* in CC is shown in **Figure 3**.

## 5 Endometrial Cancer

EC is a malignant tumor of the endometrial epithelium and one of the most common tumors of the female reproductive system (75), with 417 367 new cases and 97 370 deaths in 2020 (31). It is divided into type I, which is estrogen-related and accounts for 75%–90% of EC, and type II, which is not estrogen-related (76). Type I tumors have a good prognosis and include grade 1–2 EC, while type II malignancies include grade 3 EC, uterine serous carcinoma (USC), and clear cell carcinoma, which have a poor prognosis (77). Most EC studies suggest a role for *SOX9* as an oncogene. In EC (G1, G2, G3 tumors), atypical hyperplasia and biopsy of normal endometrial specimen tissue (proliferative and secretory stages) samples and nuclear expression of *SOX9:* (1) was usually found in the epithelial component, but not in the stromal component; (2) was significantly higher in the proliferative phase than in the secretory phase; (3) was significantly higher in the late secretory phase than the early secretory phase; (4) was significantly higher in atypical proliferative lesions than in normal and endometrial cancer tissues; (5) was significantly higher in EC tissue than normal endometrial tissue; (6) was increased significantly and progressively from G1 to G2 to G3 tumors; (7) NF-κB as well as AKT transcriptionally upregulated *SOX9* expression in EC cells, then activated the p14^ARF^/p53/p21^WAF1^ pathway, resulting in inhibition of EC cell proliferation (78). Although these findings suggest an important role for *SOX9* in endometrial cell proliferation and carcinogenesis, it cannot explain why the expression of *SOX9* in EC is higher than in normal endometrial tissue. Over-expression of *SOX9* in a mouse model induced alterations in the tissue structure of the reproductive tract in female mice and played a role in the development of histological lesions similar to endometrial polyps and hyperplasia in humans (79); endometrial hyperplasia is a precancerous lesion of EC (80). This suggests that *SOX9* is involved in the pathogenesis of endometrial diseases and may contribute to the formation of EC. *SOX9* was significantly more expressed in the UCS than in normal control endometrial tissue in USC of TCGA and GETX datasets. Meanwhile, a 4-gene (*KRT23*, *CXCL1*, *SOX9*, and *ABCA10*) signature robustly predicts OS and recurrence-free survival(RFS) of USC (81). Furthermore, the expression of both mRNA and protein of *SOX9* was significantly higher in tumor tissues than in paired adjacent tissues. The mechanism was that Circ_0109046 increased *SOX9* expression by binding miR-105, which further activated the Wnt/β-catenin signaling pathway and promoted EC cell proliferation and metastasis (82). In conclusion, upregulation of *SOX9* can promote endometrial cell proliferation, induce endometrial precancerous lesions, and predict the prognosis of EC patients in combination with the expression of other genes.

**Figure 4:**
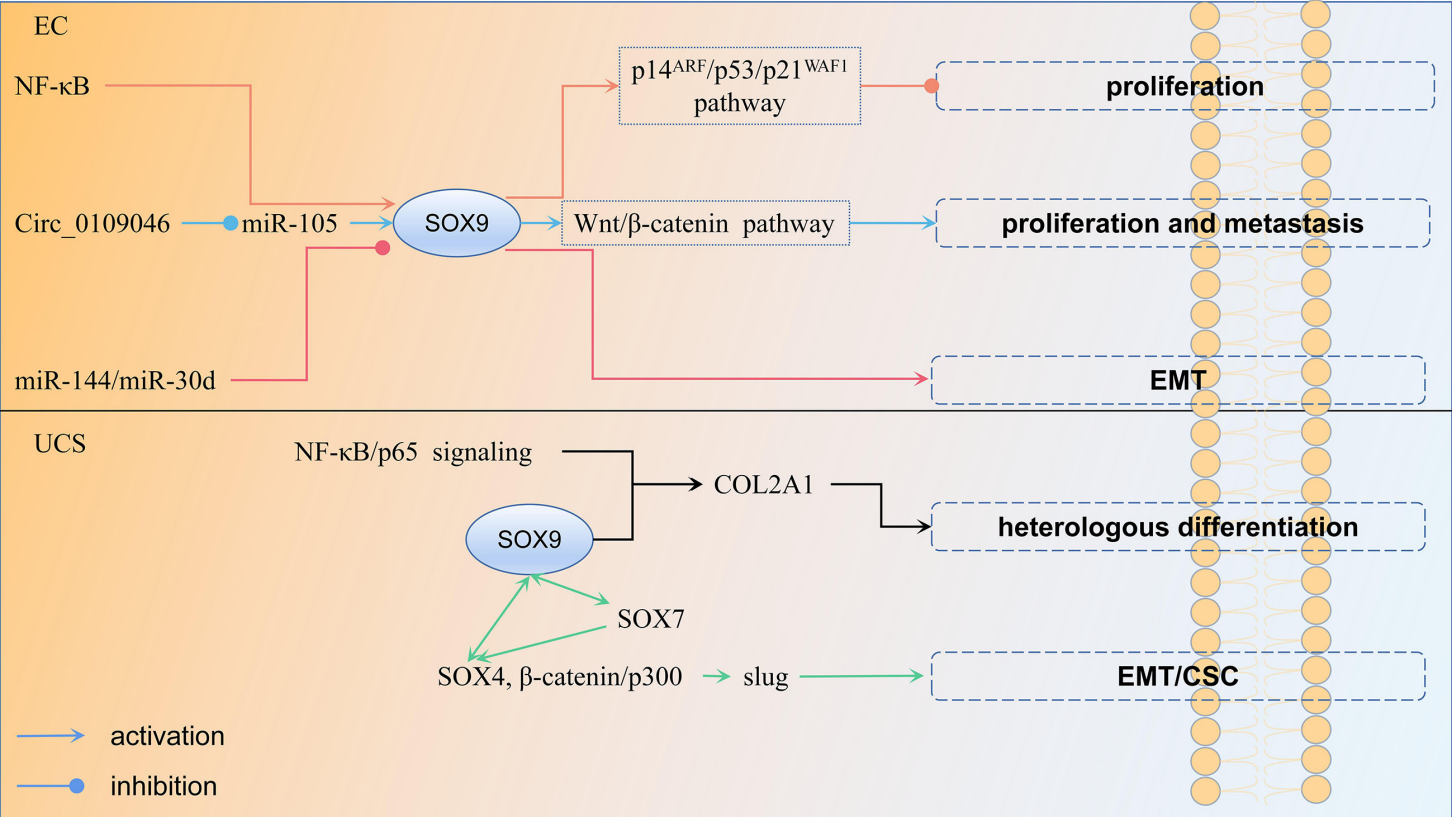
The regulation mechanism of *SOX9* in endometrial cancer and uterine carcinosarcoma each color represent a regulation mechanism of *SOX9*.

However, in the study by Opławski et al., the expression of *SOX9* was downregulated in endometrioid endometrial adenocarcinomas compared to normal endometrium (83). The possible reason for this result is the different role of *SOX9* in different EC subtypes; therefore, these roles need to be further investigated. In the same study, they used sequencing to identify three differentially expressed miRNAs, using the mirTARtool to suggest that miR-144 and miR-30d could target *SOX9 (* 83). This further suggests that miRNA regulation of *SOX9* expression leads to epithelial mesenchymal transition (EMT) development and ultimately to endometrioid adenocarcinogenesis. The regulation mechanism of *SOX9* in EC is shown in **Figure 4**.

## 6 Uterine Carcinosarcoma (UCS)

Uterine carcinosarcoma is a rare gynecological malignancy, accounting for 1%–3% of malignant tumors of the female reproductive tract (84). It is a highly aggressive, biphasic malignancy with carcinomatous and sarcomatous components (85). The former is usually glandular and includes endometrioid, clear cell, or papillary plasmacytoma, while the latter is divided into two categories, homologous (normal uterine tissue resembling endometrial mesenchymal sarcoma, smooth muscle sarcoma or fibrosarcoma) and heterologous (most commonly malignant cartilage or skeletal muscle) (86). UCS with heterologous mesenchymal stroma usually behaves more aggressively and therefore has a worse prognosis than those with homologous features. According to transformation theory, it is believed that the sarcoma component is derived from the carcinoma component through EMT (87). EMT is the process by which epithelial cells transform into an aggressive mesenchymal cell phenotype involved in invasion and metastasis of various cancer types (88). In the study of Yoshida et al., 32 UCSs were investigated. Among them, six patients with non-endometrioid carcinoma showed morphological changes in the heterologous component of the sarcoma component toward the chondrocyte phenotype. It was found that *SOX9* expression was significantly different between the two groups, and the mechanism may involve the NF-κB/p65 signaling pathway, as well as *SOX9*, contributing to the change in UCS cell morphology toward the chondrocyte phenotype by regulating *COL2A1* transcription (89). However, they did not compare the overall survival and PFS between the two groups. Therefore, the role of *SOX9* on their prognosis is unknown. *SOX9* can also cooperate with other members of its family. Increased expression of *SOX7* and *SOX9*, as well as cooperation between *SOX7* and *SOX4*, are involved in the process in which upregulation of slug by *SOX4*, β-catenin/p300 complexes induces EMT and related CSC properties, which in turn promote homologous and heterologous sarcoma components in UCS (90). In sum, upregulation of *SOX9* induces EMT, which leads to the formation of UCS, while contributing to the change of UCS cell morphology to a heterologous mesenchymal stroma, resulting in poor patient prognosis. The regulation mechanism of *SOX9* in UCS is shown in **Figure 4**.

## 7 Conclusion

(1) In OC, EC, and UCS, most studies suggest that the expression of *SOX9* in cancer tissues is higher than normal control tissues (**Table 2**) and has a certain relationship with the prognosis. This suggests that it plays an important role in these cancers and may become a new target for treatment. *SOX9* is either a tumor suppressor or an oncogene in CC, the current research conclusions are inconclusive, so it remains to be further studied. (2) *SOX9* plays an important role in gynecological precancerous lesions, carcinogenesis, development, EMT, chemotherapy resistance, maintenance of cancer stem cell properties, anti-apoptosis, migration, and invasion of cancer cells. (3) There are many mechanisms for regulating *SOX9*, including epigenetics (methylation of the promoter region); the regulation of non-coding RNA (lncRNA, miRNA, and cirRNA) (**Table 3**); and several genes. All these provide new ideas for treatment. (4) The WNT/β-catenin pathway can be activated by *SOX9* upregulation to promote carcinogenesis, chemoresistance, cell proliferation, and metastasis. (5) Whether nuclear expression or plasma expression plays a role varies by tumor subtype, but further studies are needed. (6) There is currently no study on the expression of this gene in choriocarcinoma, but the expression of its family genes has been found in germ cell tumors (91) and dissecting gonadoblastoma (92). Both tumors have choriocarcinoma components, so it can be speculated that *SOX9* is also expressed in choriocarcinoma; however, confirmation needs further study.

**Table 2:** The expression of SOX9 in gynecological malignancy.

Types of gynecological malignancy	Subtype	Expression of SOX9*	Expression site of SOX9	Role of SOX9	Referrence
OC	Sertoli-Stromal Cell Tumors	High	Unreported	Oncogene	(33)
	pure Sertoli cell tumor, endometrioid borderline tumor well-differentiated endometrioid carcinoma sertoliform endometrioid carcinoma carcinoid tumor	No comparison	Nuclear, cytoplasmic Nuclear, cytoplasmic, membrane	Oncogene	(34)
			Nuclear, membrane		
			Nuclear, cytoplasmic, membrane		
			Nuclear, cytoplasmic		
	Sertoli-Stromal Cell Tumors	No comparison	Mainly cytoplasmic, some nuclear	Oncogene	(35)
	Juvenile granulosa cell tumors	Unreported	Nuclear	Oncogene	(36)
	serous adenocarcinoma	High	Nuclear/cytoplasmic	Oncogene	(39)
	clear cell adenocarcinoma				
	Mucinous borderline				
	dysgerminoma				
	granulosa cell tumors (GCT),				
	serous ovarian carcinoma(SOC)	High	Unreported	Oncogene	(40)
	papillary serous tumors	High	Unreported	Oncogene	(41)
	serous ovarian carcinoma				
	Unreported	High	Unreported	Oncogene	(42)
	epithelial ovarian cancer(EOC)	High	Unreported	Oncogene	(43)
	high-grade serous carcinoma (HGSC).	No comparison	Nuclear/cytoplasmic	Oncogene	(44)
	Unreported	No comparison	Nuclear/cytoplasmic	Oncogene	(45)
CC	Unreported	Low	Nuclear	Suppressor	(69)
	Unreported	High	Unreported	Oncogene	(73)
	Unreported	No comparison	Mainly nuclear	Oncogene	(74)
EC	G1/G2/G3	High	Nuclear	Oncogene	(81)
	uterine serous carcinoma	High	Unreported	Oncogene	(78)
	Endometrioid endometrial adenocarcinomas	Low	Unreported	Suppressor	(83)
UCS	endometrioid	No comparison	Nuclear	Oncogene	(89)
	Non-endometrioid				
	endometrioid	No comparison	Nuclear	Oncogene	(90)
	Non-endometrioid				

*Compared to normal tissue.

**Table 3:** Studies on non-coding RNA in gynecological malignancy.

Non-coding RNA	Type	Cancer type	SOX9	Fuction	Reference
microRNA	miR-30a-5p up	OC	down	increasing sensitivity to DDP	(41)
	miR-34c up	OC	down	reducing chemoresistance	(42)
	miR-145 down	OC	up	formation of multicellular spheroids	(49)
	miR-185 up	OC	down	inhibiting OC growth and development	(50)
	miR-130a up by*	CC	up	promoting chemoresistance to DDP	(72)
	miR-215-3p up	CC	down	inhibiting growth and metastasis	(73)
	miR-144 up miR-30d down	EC	down	leading to Epithelial mesenchymal transition(EMT)	(83)
lncRNA	LINC00115 binds to miR-30a	OC	up	Enhancing stemness of CSCs and preventing apoptosis	(57)
	LINC01132 binds to miR431-5p	OC	up	increasing migration and invasion	(43)
	LINC00284-miRNA-195/497-5p-mRNA by*	OC	up	promoting tumor initiation and progression	(40)
circular RNAs	Circ_0109046 binding to miR-105	EC	up	promoting proliferation and metastasis	(82)

*Represents regulation by SOX9.

SOX9 in different gynecologic tumor cell lines after being regulated by non-coding RNA.

## Author Contributions

All authors listed have made a substantial, direct, and intellectual contribution to the work and approved it for publication.

## Funding

This work was supported by Effect of neoadjuvant chemotherapy with paclitaxel and carboplatin combined with intraperitoneal perfusion of bevacizumab on prognosis of advanced ovarian cancer [grant numbers S2021SFYLJS0026].

## Conflict of Interest

The authors declare that the research was conducted in the absence of any commercial or financial relationships that could be construed as a potential conflict of interest.

## Publisher’s Note

All claims expressed in this article are solely those of the authors and do not necessarily represent those of their affiliated organizations, or those of the publisher, the editors and the reviewers. Any product that may be evaluated in this article, or claim that may be made by its manufacturer, is not guaranteed or endorsed by the publisher.

## Abbreviations

OC, ovarian cancer; CC, cervical cancer; EC, endometrial cancer; HGM, high migration group; CSCs, cancer stem cells; ORF, open reading frame; NLS, nuclear localization signal; NES, nuclear output signal; SCST, Sex-cord stromal tumors; SSCTs, Sertoli-Stromal Cell Tumors; GCT, granulosa cell tumors; PFS, Progression-Free-Survival; OS, Overall survival; MCS, multicellular spheroids; OCSCs, ovarian cancer stem cells; EOC, epithelial ovarian cancer; SOC, serous ovarian carcinoma; SNPs, nucleotide polymorphisms; LCR, long control region; EMT, Epithelial mesenchymal transition; EEC, endometrioid carcinoma; USC, uterine serous carcinoma; UCS, Uterine carcinosarcoma.
